# Review of Experimental and Clinical Studies of Bovine Foetal Collagen Application

**DOI:** 10.1111/wrr.70213

**Published:** 2026-11-06

**Authors:** Jordan Johnson, Daniella King, Mehul Satish Prabha, Claudy Sarpong, Barite Gutama, Franklin Gergoudis, Emmanuel Giannas, Rachel Walden, Wiliam C. Lineaweaver

**Affiliations:** ^1^ Department of Plastic Surgery Vanderbilt University Medical Center Nashville Tennessee USA; ^2^ Vanderbilt University School of Medicine Nashville Tennessee USA; ^3^ Meharry Medical College Nashville Tennessee USA; ^4^ Loma Linda School of Medicine Loma Linda California USA; ^5^ Eskind Biomedical Library Vanderbilt University Nashville Tennessee USA

## Abstract

Soft tissue defects continue to present a significant clinical challenge in plastic and reconstructive surgery. Tissue substitutes, such as dermal matrices, have been used in wound care and reconstructive practices. Specifically, foetal bovine collagen (FBC) based dermal matrices possess unique collagen profiles when compared to other scaffolds. Despite increased use in wound healing, the literature evaluating outcomes and efficacy of FBC remains insufficient. We reviewed the available evidence on FBC‐based dermal scaffolds in clinical and preclinical studies and conducted this review in accordance with PRISMA guidelines. The search strategy was developed in collaboration with our institution's medical librarians, and the final search was completed in four databases on November 20, 2025. Clinical, ex vivo and in vivo investigations published between 2006 and 2025 that evaluated FBC were eligible for inclusion. A narrative synthesis was performed due to substantial heterogeneity in outcome reporting. Of the 1132 initially identified studies, 28 met the inclusion criteria. Outcomes included wound healing, time to healing, biologic responses and physical characteristics. Overall, FBC‐based dermal matrices demonstrated regenerative potential across varying indications, with favourable wound closure outcomes relative to standard of care and untreated controls, though findings were inconsistent across comparators. Though promising data exists, heterogeneity in outcome reporting and study design limits substantiated conclusions; this prevents clinicians from making evidence‐based decisions regarding use of FBC.

## 1 Introduction

Soft tissue defects arising from trauma, oncologic resection, chronic wounds, burns and reconstructive procedures remain a frequent and complex challenge in plastic and reconstructive surgery. Chronic wounds alone affect millions of patients in the United States and are associated with substantial morbidity, health care utilization and economic burden, with annual treatment costs estimated to exceed $25 billion [1, 2]. Current reconstructive options for soft tissue loss include autologous skin grafts, local and free tissue transfer, synthetic meshes and acellular dermal matrices (ADMs). These techniques have broadened reconstructive possibilities but carry inherent limitations. Autologous reconstruction may be subject to donor site morbidity, limited tissue availability and additionally often require microsurgical expertise [1, 2]. In contrast, the use of synthetic and biologic materials can lead to infection, foreign body response, delayed incorporation and variable long‐term outcomes [3, 4, 5].

In response to these limitations, biologic scaffolds were developed to facilitate wound healing through host mechanisms. These scaffolds promote tissue regeneration while eliciting an anti‐inflammatory immune response, decreasing the risk of foreign body reactions that occur with synthetic scaffolds [6]. ADMs are now widely used across reconstructive and wound care applications; however, their clinical performance is heterogeneous and influenced by factors such as tissue source, processing techniques and collagen architecture [7, 8, 9, 10].

### 1.1 Overview of Foetal Bovine Collagen as a Biologic Scaffold

The biologic behaviour of FBC is closely related to its collagen composition. In adult skin, the dermis and epidermis contain collagen Types I–V, with the dermis forming irregular connective tissue made up of interwoven collagen and elastic fibres [11]. Whereas the foetal dermis develops from a highly cellular mesenchyme into a predominantly collagenous connective tissue with papillary and reticular layers [11].

A distinct feature of foetal dermis is its high proportion of Type III collagen to Type I collagen, which persists at levels greater than that of adult skin [12, 13]. As the foetus develops, Type I collagen increases compared to Type III, but still, foetal Type III collagen content exceeds that of adult dermis. Type III collagen is associated with increased matrix flexibility, enhanced cellular migration and reduced intermolecular collagen crosslinking [14]. These characteristics promote tissue regeneration, rather than scar formation, making FBC an ideal scaffold for reconstructive cases [15, 16].

Beyond collagen composition, FBC has a “flexi‐rod” architecture made up of Type III collagen that is fundamental to its tissue regeneration capability. This extracellular matrix‐like network not only facilitates cellular infiltration but also provides flexible networks that are typical of skin, whereas Type I collagen typically produces thicker, more organized fibrillar structures [15, 17, 18]. Decreased crosslink density in foetal bovine matrices supports host remodelling and adaptation after implantation.

The biologic response of foetal bovine dermis–derived products has been described as an “adoption and adaptation” process, distinct from the encapsulation or rapid resorption observed with other extracellular matrix biomaterials [19]. Histologic evaluation demonstrated minimal inflammatory response, rapid cellular repopulation and early vascularization following implantation. Importantly, FBC scaffolds persisted as metabolically active tissue and exhibited structural reorganization in response to mechanical loading, including collagen fibre realignment in mechanically active environments such as tendon repair (Figure 1). This behaviour contrasts with crosslinked matrices, which frequently elicited fibrous encapsulation and small intestinal submucosa–derived products, which underwent rapid degradation accompanied by pronounced inflammatory responses.

**FIGURE 1:**
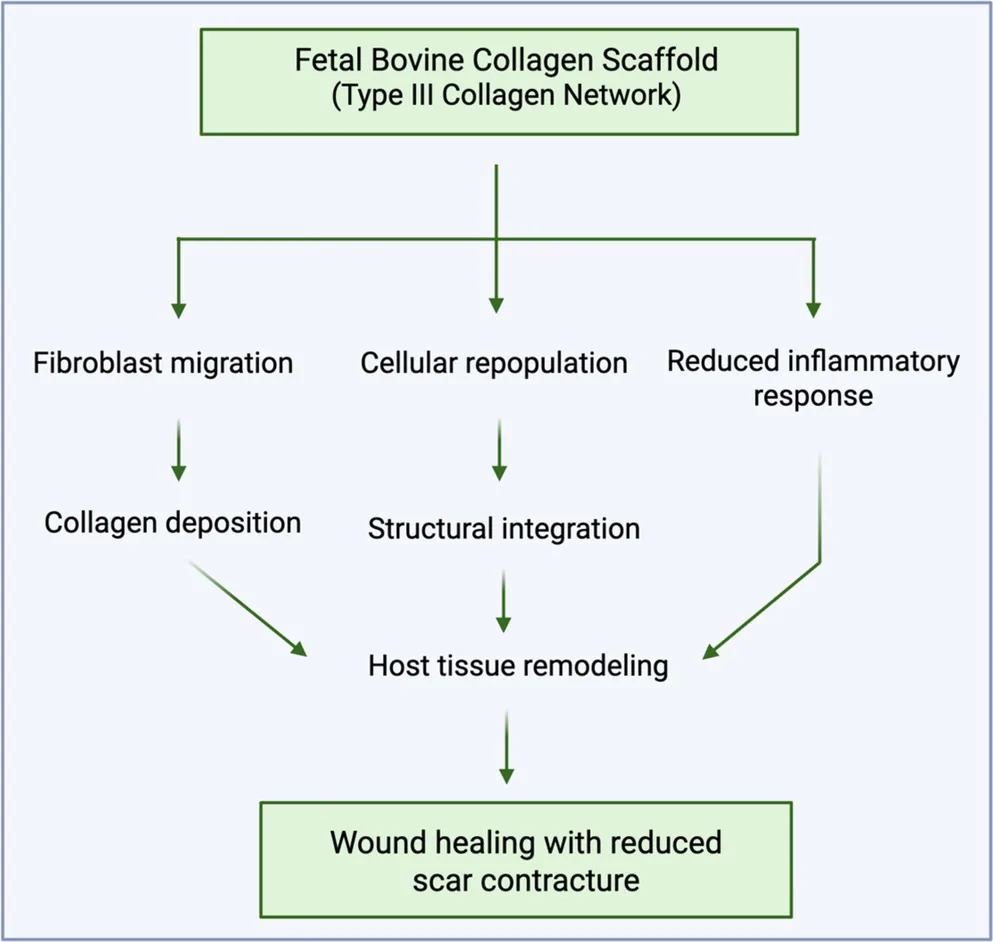
Summary of mechanism of foetal bovine collagen in wound healing, synthesizing findings from both preclinical and clinical studies. Created in BioRender (2026).

FBC scaffolds are commercially available in a variety of formats and configurations, including thick and thin sheets as well as meshed and non‐meshed constructs [20]. This variety allows surgeons to optimize outcomes by tailoring scaffold selection to wound characteristics. Thickness and presence of mesh influence conformability, fluid egress and integration with surrounding tissue. Typically, thinner or meshed constructs facilitate improved contouring and incorporation in wound beds [21, 22]. Various scaffold designs support the use of FBC across a wide range of reconstructive and chronic wound cases.

Despite these advantages, literature evaluating the use of FBC‐based dermal scaffolds remains fragmented. Existing systematic reviews fail to exclusively evaluate FBC, usually grouping diverse ECM products together, leaving isolated reports on FBC. This lack of synthesized literature limits the ability of surgeons to efficiently evaluate and make evidence‐based decisions regarding scaffold selection for wound healing and reconstructive indications. In order to consolidate the current data, identify gaps in the literature and guide future research and clinical application, this systematic review aims to assess the clinical and preclinical evidence on FBC‐based dermal scaffolds in wound healing and reconstructive surgery.

## 2 Methods

This systematic review was conducted in accordance with the Preferred Reporting Items for Systematic Reviews and Meta‐Analyses (PRISMA) guidelines [23]. The study protocol was registered with the International Prospective Register of Systematic Reviews (PROSPERO; CRD420251234851). A search strategy was developed in coordination with institutional librarians. A systematic search of PubMed, Embase, Web of Science and the Cochrane library was completed on November 20, 2025 for relevant studies.

Study screening was independently performed by M.P. and D.K., with discrepancies resolved by J.J. and escalation to W.L. when necessary. All records were managed and screened using Covidence systematic review software (Veritas Health Innovation, Melbourne, Australia).

Studies published between 2006 and 2025 that evaluated FBC were eligible for inclusion. Eligible study designs included investigations involving human subjects aged 18 years or older, animal studies, ex vivo studies and in vitro studies. Both studies evaluating FBC in isolation and those comparing FBC to other biologic scaffolds were included. Systematic reviews, narrative literature reviews and conference abstracts were excluded. Publications focused on non‐dermal extracellular matrix materials or non‐foetal bovine sources were also excluded. Studies reporting outcomes for other membranes or ADMs were included only if FBC served as a comparator.

Extracted variables included author, year of publication, country of origin, study design, FBC formulation, fixation method, comparator characteristics, reported outcomes (histologic and clinical) and complications. We completed a narrative synthesis due to the heterogeneity of study design, interventions and outcome measures. Majority of studies (21/29) were non‐comparative observational designs, preventing pooling of results and meta‐analysis. Risk of bias was assessed independently by two reviewers, with disagreements adjudicated by a senior author. Risk of bias assessment was conducted according to study design. Randomized controlled trials (RCTs) were evaluated using the Cochrane Risk of Bias Tool (RoB 1), non‐randomized comparative clinical studies were assessed using the ROBINS‐I tool and non‐comparative clinical studies were evaluated using the SYRCLE risk of bias tool [24, 25, 26]. In vivo and ex vivo studies were assessed using a custom quality assessment checklist adapted for laboratory‐based research. Funding source and disclosed author and industry relationships were recorded for each study when reported and considered in our risk of bias assessment.

## 3 Results

The search yielded a total of 1132 studies across the included databases: Embase—521, Web of Science—330, PubMed—260, Cochrane—21. After removing 403 duplicates, 729 studies were screened. Based on title and abstract screening, 680 studies were deemed irrelevant and 49 studies were assessed for inclusion by full text review. A total of 28 studies met the aforementioned criteria; 21 were removed for reasons including wrong intervention (*n* = 6), wrong outcomes (*n* = 6), wrong study design (*n* = 8) and full text not accessible (*n* = 1) (Figure 2).

**FIGURE 2:**
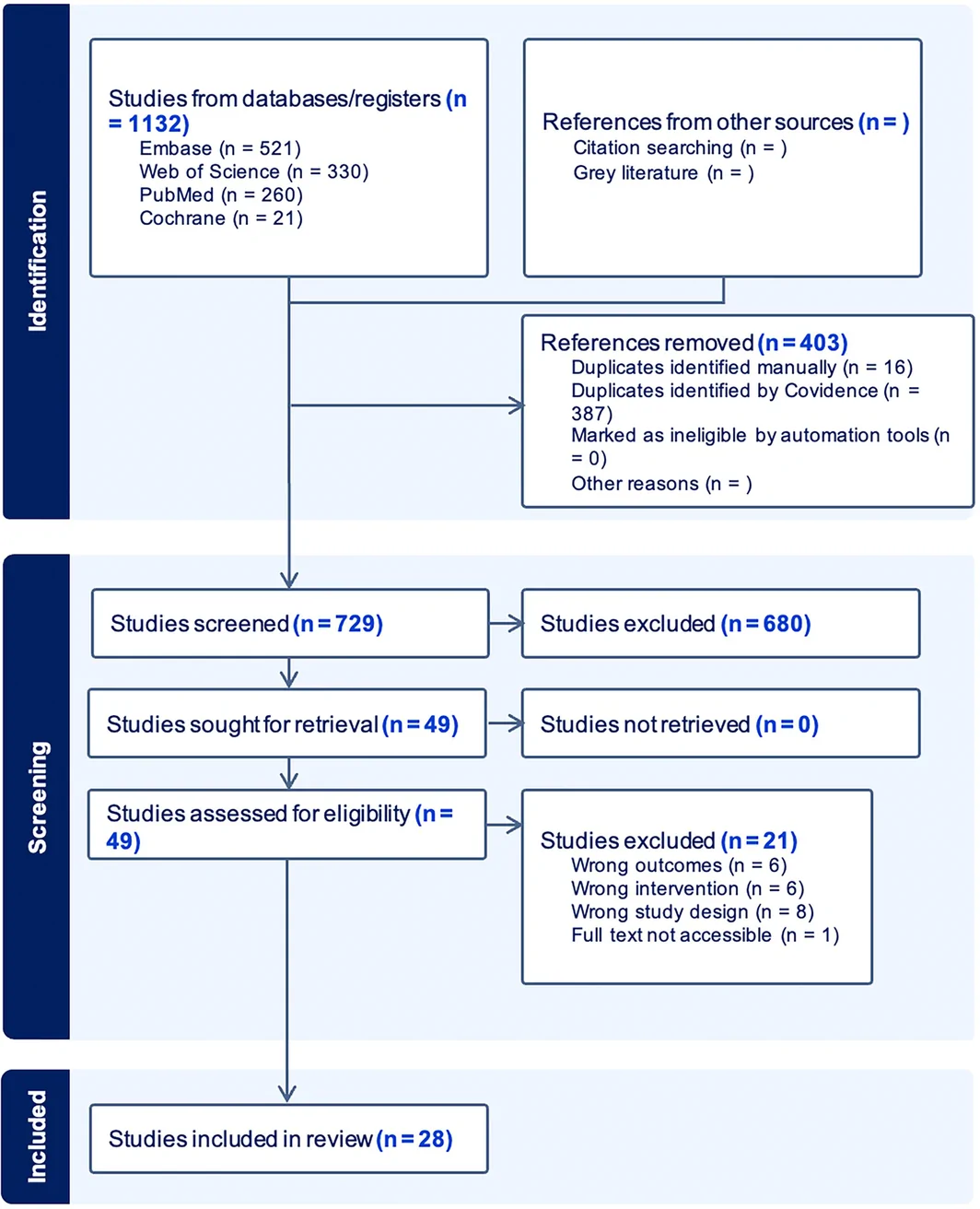
PRISMA flow diagram of study selection.

A total of 28 studies were included in the final analysis with publication dates ranging from 2008 to 2024. Majority of the studies were conducted in the United states (*n* = 22), with others in China (*n* = 2), Iran (*n* = 2), New Zealand (*n* = 1) and Italy (*n* = 1). Study design varied with retrospective cohort studies (*n* = 9), prospective cohort studies (*n* = 3), case reports (*n* = 2), case series (retrospective/prospective) (*n* = 5), preclinical studies (*n* = 8) and randomized controlled trials (*n* = 1).

Overall, 28 studies were included; 19 were clinical and 9 were preclinical. Among the clinical studies, FBC was evaluated for chronic wound healing in adults in 13/19 studies. Diabetic foot ulcers (DFU) were the most common indication in 6/19 studies with venous leg ulcers/venous stasis ulcers being used in 2/19 studies, pressure injuries in 1/19 studies, 1 study including chronic lower extremity wounds/limb salvage wounds and 5 studies did not limit the wound aetiology to one indication. Other clinical applications included burn wounds, which were reported in 2/15 studies, deep/partial‐thickness wounds in 1 study, breast reconstruction/tissue expander support in 1 study and periodontal root coverage in 1 study. Some studies included multiple wound causes, and as a result, counts may overlap. Of the 9 preclinical animal wound/healing models and biomaterials/cellular assays, 3 were in vivo/in vitro studies, 5 were in vivo‐only studies, 1 was an in vitro‐only study and 1 was a randomized controlled animal study.

The FBC Interventions were mostly delivered as sheets or membranes, which occurred in 21/22 studies, with one study evaluating an FBC scaffold. Among the clinical studies, FBC was used as a stand‐alone wound dressing in 9/15 studies. The remaining 6/15 studies used additional wound‐care measures including silicone contact layers, negative pressure wound therapy (NPWT), compression therapy and/or autografting in selected patients.

Of the 28 studies included, the majority were assessed as having some concerns for risk of bias (*n* = 24), while three were judged to have low risk (*n* = 3) and one was determined to have high risk of bias (*n* = 1). Among cohort studies, primary concerns centred on confounding, participant selection and outcome measurement. Case series frequently lacked clear outcome reporting and adequate follow‐up. In animal studies, identified risks were largely related to insufficient blinding of caregivers and outcome assessors. Similarly, in vitro and ex vivo studies often did not adequately describe statistical methods used for outcome analysis. Finally, the RCT was assessed as having some concerns for bias due to the inability to blind investigators.

### 3.1 Experimental Studies

This review comprised nine experimental studies, a combination of in vivo and ex vivo models, with a range of reported outcomes.

#### 3.1.1 Structural Interactions

Experimental studies assessed interactions between the host and FBC scaffold, with an emphasis on collagen architecture, content and integrity. Cook et al. evaluated healing and tissue regeneration in rats with abdominal wall defects and found non‐crosslinked foetal bovine collagen (NCLFBC) to have limited cellular infiltration and modest tissue integration by 12 weeks—poorer remodelling than cross‐linked and non–cross‐linked porcine and bovine dermal matrices [27]. Cornwell et al. reported that collagen fibre architecture was shown to evolve from crosshatch to aligned fibre patterns when mechanically stimulated and found scaffold collagen interwoven with host connective tissue, indicating successful integration [19]. Wang et al. analysed FBC protein composition and found predominance of Type I and III collagen, with little retention of other ECM proteins and absence of associated growth factors; Heal‐All–type matrices lacked preserved ECM interaction networks and functioned primarily as structural collagen scaffolds, whereas SurgiMend retained a broader, though limited, ECM protein profile relative to human and porcine‐derived matrices [28]. These findings suggest that FBC integrates into host tissue gradually, with structural remodelling and protein retention varying by comparator matrix.

#### 3.1.2 Mechanical Properties

Experimental studies also investigated scaffold mechanical properties after implantation. Mansour et al. found that enzymatically decellularized foetal acellular dermal matrix (FADM) tensile strength (1.07 ± 0.07 MPa) was significantly lower compared to standard FADM (2.5 ± 0.85 MPa) and calf acellular dermal matrix (CADM) (5.9 ± 0.72 MPa). Also, Young's modulus of CADM was approximately 21.5 times greater than that of FADM's (*p* < 0.0001) [29]. Cornwell et al. compared incisional hernia repair reinforced with FBC to component repair alone in a rat model and found FBC reinforced repairs improved strength and significantly reduced hernia formation at timepoints up to 1 year [30].

Adelman et al. investigated the effect of different sutures on FBC integration in a rat model. Attachment strength ranged from 2.13 ± 0.93 N (Vicryl Rapide) to 15.23 ± 2.82 N (biologic attachment) at 4 weeks; while at 12 weeks strength ranged from 2.89 ± 1.48 N (Maxon) compared to 12.88 ± 5.93 N (biologic attachment) [31]. In summation, these findings show that FBC's mechanical performance is weaker than FADM and CADM, but improves with reinforcement.

#### 3.1.3 Tissue Ingrowth

Adelman et al. assessed cellular repopulation of FBC scaffolds of various suturing methods in rats and found infiltration localized to the anterior third of the implant closest to the abdominal wall for all sutures (degradable polymer and permanent polypropylene) except the biologic suture [31].

#### 3.1.4 Cellular and Inflammatory Response

As a foreign material, FBC may elicit a host inflammatory response following in vivo implantation. Agostinis et al. compared FBC to Integra and PELNAC dermal matrices and found lower endothelial cell colonization, proliferative stimulation and angiogenic potential in both in vitro and in vivo assays [32]. Also, Feng et al. found that FBC supported neuronal differentiation of human bone marrow stem cells, with cultured cells developing neuron‐like morphology, forming multilayered network structures and expressing βIII‐tubulin [33]. Cornwell et al. similarly found that FBC revascularized and repopulated host cells within 2 weeks of placement in a rat incisional hernia model [30]. Lastly, Mansour et al. reported a decrease in inflammatory response following implantation of FADM compared to controls [34, 35]. These findings show that FBC generates a relatively mild host response—weaker than Integra and PELNAC specifically, but still able to support cellular repopulation, revascularization and decreased inflammation.

#### 3.1.5 Remodelling and Matrix Maturation

Rennert et al. reported increased collagen density and progressive collagen organisation by postoperative Day 28 in mice wounds treated with FADM compared with untreated controls [30, 35].

#### 3.1.6 Functional Outcomes in Experimental Models

Cornwell et al. induced ventral incisional hernias with anterior separation in rats, and showed that dense connective tissue persisted for 1 year [30]. Mansour et al. compared preparation methods of FADM in a full‐thickness rat model: ethylene oxide–sterilized, enzymatically decellularized and gamma‐irradiated FADM each demonstrated high rates of closure(97%, 97% and 96.6%, respectively) compared with 60.1% without an ADM [29]. In another study, Mansour et al. evaluated the effect of FADM with human Wharton's jelly mesenchymal stem cells (hWJ‐MSCs) for full‐thickness skin wounds in rats, with wound healing rates of FADM alone (91.6%), FADM with hWJ‐MSCs (100%) and no product (61.1%) [34]. In summation, these findings support that FBC‐based scaffolds support durable structural outcomes under mechanical stress, achieve high wound closure rates and show further improvement in conjunction with stem cell therapy.

### 3.2 Clinical Studies

A total of 19 clinical studies evaluated FBC across a variety of indications, ranging from diabetic foot ulcers to breast reconstruction (Table 1, Figure 3). Primary outcomes most commonly included time to healing, although several studies reported alternative outcome measures (Table 1).

**TABLE 1:** Summary of available evidence.

Wound aetiology	Author, year	Design (*n*)	Wound type	Comparator	Primary outcome(s)
Chronic wounds	Fitzgerald, 2019	Retrospective Cohort (208)	Burn	Human fibroblast derived dermal substitute (HFDS)	25 weeks to wound healing
	Gormley, 2024	Prospective Cohort (22)	DFU + limb threatening ischemia	None	178 days to wound healing
	James, 2022	Retrospective Cohort (252)	DFU + venous stasis ulcers	Saline dressing	Need for amputation, OR: 0.433
	Karr, 2011	Retrospective Cohort (48)	DFU	Apligraf	37 days to healing
	Lantis, 2021	Randomized Controlled Trial (207)	DFU	Standard of care	57 days to healing
	Lullove, 2023	Prospective Cohort (20)	DFU	None	7.85 weeks to healing
	Sabolinski, 2018	Retrospective Cohort (927)	Venous leg ulcers	Apligraf	Time to wound closure: Apligraf (19 days), FBCD (30 days)
	Sabolinski, 2024	Retrospective Cohort (1182)	Pressure injuries	Apligraf	Time to wound closure: Apligraf (103 days), FBCD (162 days)
Acute Wounds	Bergus, 2024	Retrospective Cohort (173)	Burn	None	84.4% complete wound closure
	Parcels, 2014	Case Report (1)	Burn	None	Wounds completely closed
	Strong, 2016	Case Report (1)	Burn	None	Complete wound closure Day 80–102
Surgical wounds/resections	Lineaweaver, 2012	Retrospective Case Series (15)	Abdominal hernia	None	93% repair success
	Gaster, 2013	Prospective Cohort (12)	Breast reconstruction	None	Limited inflammation and neovascularizaition
Traumatic/mixed aetiology wounds	Hayn, 2014	Retrospective Cohort (38)	Not limited	Addition of NPWT	Time to healing 91.4 days (FBC), 140.3 days (FBC + NPWT)
	Neill, 2012	Prospective Case Series (7)	Not limited	None	100% graft integration
	Neill, 2013	Retrospective Case Series (9)	Not limited	None	Histological tissue response, descriptive
	Lineaweaver, 2015	Prospective Case Series (5)	Not limited	Uninjured human dermis	Suppression of αSMA in FBC occupied areas
	Strauss, 2012	Retrospective Cohort (49)	Not limited	None	75.9% wound closure
Other applications	Naud, 2020	Case Series (8)	Gingival recession/periodontal	None	85.96% root coverage

*Note:* Concise summary of included studies including wound aetiology, author, year, study design, sample size, wound type, comparator, and primary outcomes.

Abbreviation: NPWT, negative pressure wound therapy.

**FIGURE 3:**
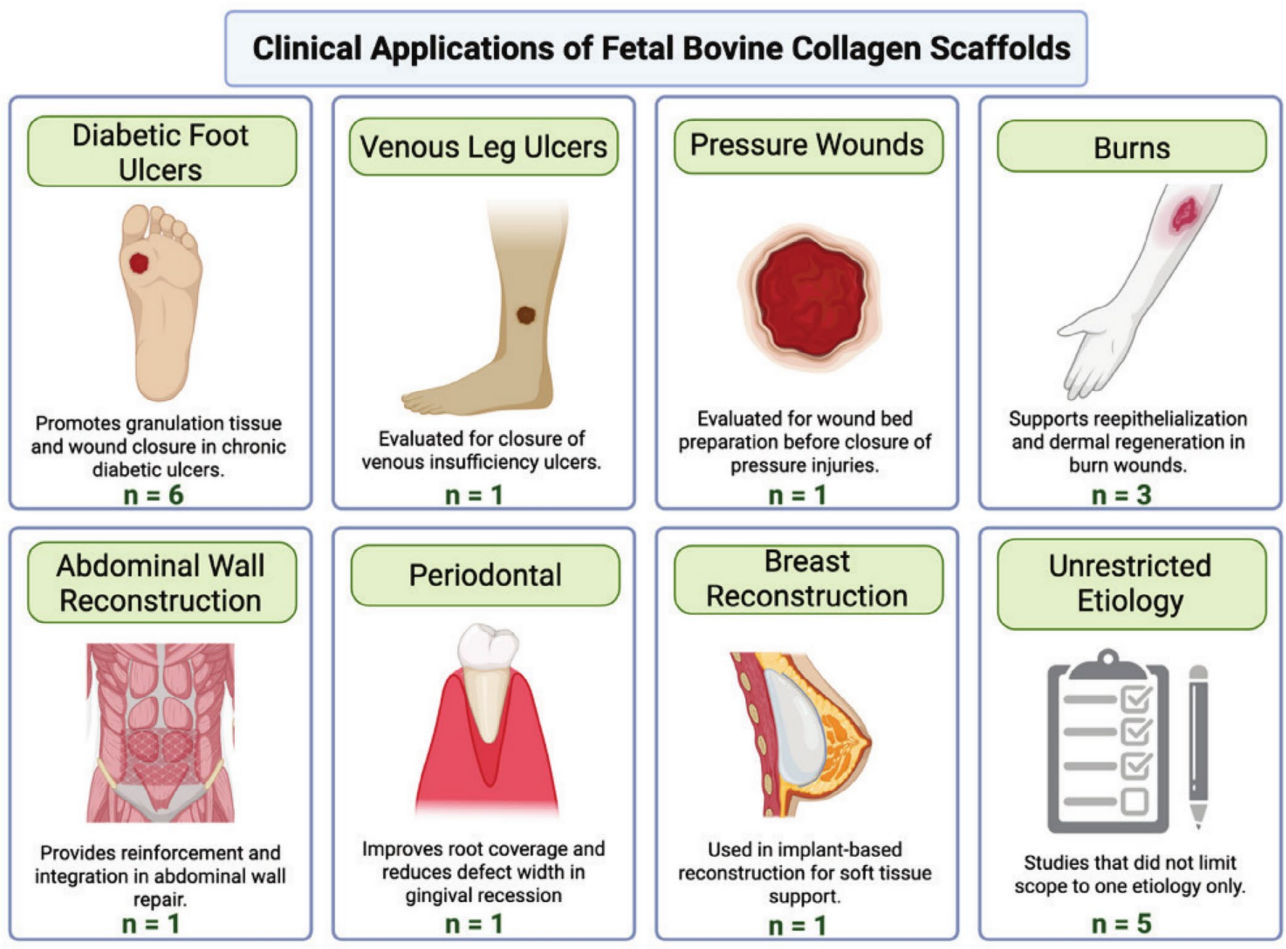
Clinical applications of FBC by wound aetiology. The presented categories reflect the 19 clinical studies included in this manuscript in Table 1. Created in BioRender (2026).

#### 3.2.1 Tissue Ingrowth

Gaster et al. evaluated tissue expander based breast reconstruction with FADM and found that cellular infiltration ranged from grade zero to three, with greater incorporation at the interface with native host tissue [36].

#### 3.2.2 Cellular and Inflammatory Response

Gaster et al. also determined host inflammatory response and capsule formation after FBC implantation. In 12 patients, histologic analysis found minimal inflammatory cells at 23 months and an acellular capsule at the interface of FBC with the tissue expander at explanation. Also reported limited neovascularization overall that was more prominent when SurgiMend was placed beneath well vascularized mastectomy flaps [36, 37].

Neill et al. evaluated the biological response to FBC implantation through histologic analysis of biopsies obtained from patients with full‐thickness wounds at the second stage of skin grafting. Early responses (11–18 days) demonstrated fibroblast infiltration, neovascularization and granulation tissue formation; intermediate responses (19–27 days) showed increased fibroblast density within the matrix with less prominent granulation tissue; and late responses (9 months) were characterized by matrix remodelling with fibroblast proliferation, a basket‐weave collagen pattern, absence of scar contracture and stable skin graft adherence. This temporal progression mirrored normal wound healing. Overall, FBC was well tolerated, with minimal inflammatory response and consistent neovascularization extending from the edges of the implant [37].

These findings show that FBC was well tolerated across different clinical scenarios, creating a mild inflammatory response and cellular response consistent with normal healing, but the neovascularization may be more dependent on the characteristics of the implantation site.

#### 3.2.3 Replacement With Host Tissue

As a part of evaluating the wound healing progress, some clinical studies reported replacement of the matrix with host tissue. Specifically, Gaster et al. reported slow, partial replacement by host collagen with the replacement occurring primarily at fenestrations. Ultimately, they reported incomplete host collagen replacement over long implementation periods [36, 38].

#### 3.2.4 Wound Contracture

Studies also examined wound contracture following FBC placement. Lineaweaver et al. evaluated full thickness wounds treated with FBC and excised samples during the second stage of split‐thickness skin graft, assessing for the presence of *α* smooth muscle actin. The authors found that areas treated with FBC were associated with lower α smooth muscle actin expression than in uninjured tissue [38].

#### 3.2.5 Chronic Wounds

The majority of included clinical studies evaluated the use of FBC in chronic wounds with outcomes related to reepithelialization, granulation tissue formation, wound healing time and rates of complete closure. Strong et al. reported a case of a 48‐year‐old woman with 25% total body surface area burns treated with FBC, achieving reepithelialization by Day 26 and wound closure between Days 80–102 [39]. Parcells et al. presented a 71 year‐old woman with full‐thickness burns of both hands treated with FBC that demonstrated [40] granulation tissue by Day 5 with eventual would closure [40]. In a cohort study, Bergus et al. reported an 84.4% complete wound closure rate using PriMatrix alone in 173 paediatric patients with deep partial‐ and full‐thickness burns [41].

Strauss et al. evaluated difficult‐to‐heal wounds of mixed aetiologies with an overall healing rate of 75.9% (63.8% reepithelialization, 12.1% requiring skin grafting), with 65.5% reepithelialized by 12 weeks and 86.2% by 15 weeks [42].

In studies of diabetic foot ulcers (DFUs), Lantis et al. conducted the only RCT, enrolling 207 patients with DFUs treated with either FADM or the standard of care. They found a higher rate of wound closure with FADM (45.6% vs. 27.9%) but a longer median time to closure (57 vs. 43 days) compared to the standard of care [42]. Fitzgerald et al. reported that a human fibroblast–derived dermal substitute demonstrated a 77% greater probability of healing compared with FBC dressing [43].

Several studies reported time to wound healing as a primary outcome. Karr et al. compared PriMatrix and Apligraf in 48 patients with DFU or venous stasis ulcers [44], and found a shorter time to healing with PriMatrix for both DFU (37.5 ± 23.2 vs. 86.8 ± 81.7 days) and venous stasis ulcers (31.5 ± 18.9 vs. 62.9 ± 32.6 days) [45]. Lullove et al. reported a mean time to healing of 7.85 weeks for hard‐to‐heal full‐thickness lower extremity DFUs, with all wounds healed by Week 15 [44]. Gormley et al. reported a median time to healing of 178 days in 22 patients with difficult‐to‐heal chronic foot ulcers, with substantial variation by WIfI stage (105 days for Stage 3 vs. 219 days for Stage 4) [46].

Larger cohort studies compared FBC against similar products [43]. Sabolinski et al. evaluated 927 patients with venous leg ulcers and reported a median time to wound closure of 19 weeks with bilayered living cellular constructs compared with 30 weeks using FBC dressing (*p* = 0.01) [47]. In a separate cohort of 1182 patients with Stage II–IV pressure injuries, the same group reported faster wound closure time with BLCC than FBC (103 vs. 162 days; *p* < 0.0001) [48].

Hayn et al. evaluated 38 wounds with partial‐ or full‐thickness traumatic and surgical wounds and reported a shorter mean time to healing with PriMatrix alone compared with PriMatrix combined with negative pressure wound therapy (91.4 vs. 140.3 days) [49].

#### 3.2.6 Staged Skin Grafting

Neill et al. evaluated the use of FBC as a dermal substitute in two‐stage split‐thickness skin graft procedures for wounds of various aetiologies. In their evaluation of seven patients, they found that FBC provided sufficient structural stability in the interval period between procedures, and all patients ultimately had successful skin grafts following the staged reconstruction [20].

#### 3.2.7 Breast Reconstruction

Gaster et al. demonstrated that FBC can be used in tissue expander based breast reconstruction and showed no failures attributable to the matrix [36].

#### 3.2.8 Abdominal Wall Reconstruction

Lineaweaver et al. evaluated FBC in abdominal wall defects, and found successful reconstruction in 14/15 (93%) patients with midline abdominal wall separation, which is significantly better than standardized component repair [50].

#### 3.2.9 Limb Salvage

James et al. evaluated the effects of FBC on limb salvage in patients that received debridement with use of cellular and tissue based products; 109 patients of the total 252 patients were treated with FBC. FBC was associated with a decrease in the need for future amputation relative to other treatments (OR: 0.433, 95% CI: 0.193–0.970, *p* < 0.05) [51].

All of the aforementioned studies show the healing ability of FBC across DFU, venous leg ulcer, pressure injuries and burn populations, with varying healing rates and time to closure varying by wound type, patient population and study design. In comparative studies, the data are mixed—FBC outperformed standard of care and Apligraf, but underperformed a human fibroblast derived substitute and no comparator was favoured consistently across studies.

### 3.3 Risk of Bias Assessment

Risk of bias assessment was completed with the appropriate tool based on study design. Of the 28 studies included, 12 (43%) disclosed a financial relationship with an industry sponsor. Six were affiliated with TEI Biosciences (manufacturer of PriMatrix), and two studies reported author consulting or employment relationships [31, 32, 34, 38, 47, 48]. Two studies were funded by Integra (manufacturer of SurgiMend), one in which the statistics were completed by the company. Two were funded by Organogenesis, manufacturer of a competing scaffold evaluated against FBC [44, 45]. One study lacked a conflict of interest section and therefore could not be properly evaluated for bias. Per our assessment, 24 studies were decided to have some risk of bias, 3 were found to have low risk of bias and 1 study was determined to have high concerns for bias.

## 4 Discussion

### 4.1 Principal Findings

This systematic review identified 28 studies evaluating FBC based dermal scaffolds in various clinical and preclinical conditions. The evidence synthesized supports the biologic capability of FBC use in wound healing and reconstructive applications. Despite the encouraging evidence for FBC use, the literature remains limited: most studies are retrospective cohort studies or small case series, with only one RCT. As a result, definitive conclusions regarding efficacy, indications and comparative performance of FBC scaffolds relative to existing alternatives are difficult to establish. The following sections interpret the evidence by wound aetiology, since the strength of evidence differs across wound types.

### 4.2 Interpretation by Wound Aetiology

#### 4.2.1 Diabetic Foot Ulcers and Chronic Lower‐Extremity Wounds

DFUs are the most heavily studied indication for FBC, including the only included RCT. Though the most evaluated indication, the comparative picture remains mixed; in some cases, FBC outperformed the standard of care and a competing scaffold while underperforming when compared to an alternative dermal substitute in another. The conflicting evidence and the lack of multiple RCTs show that DFU is the indication with the most evidence, yet there is still no clear verdict.

#### 4.2.2 Venous Leg Ulcers and Pressure Injuries

The two largest cohort studies included in this review come to a common conclusion: a bilayered living cellular construct had a shorter time to healing than FBC. This is the most direct evidence on FBC clearly being outperformed rather than understudied. However, it is necessary to note that both studies were funded by the manufacturer of the “superior” comparator, not FBC. By no means does this negate the findings, but does indicate that the conclusion is built on sponsor‐funded data with no independent study to compare against.

#### 4.2.3 Burn Wounds

Evidence regarding FBC use in burn wounds remains mostly descriptive—two case reports showing favourable outcomes, but no comparator arms and only a single cohort study. The literature provides the least evidence to support a clinical recommendation for use in burn wounds at this time, and is in need of more data before any clinical claims can be made.

#### 4.2.4 Surgical and Reconstructive Applications

The procedures covered include: staged grafting, breast reconstruction, abdominal wall repair and mixed traumatic/surgical wounds. The evidence supporting these indications is smaller studies that are uncontrolled and descriptive. They are useful only for proof of concept but lack a comparative model to determine the performance of FBC with the current standard of care. Each of the included indications in this section could benefit from cohort‐level comparative data before it can be considered as an alternative treatment.

### 4.3 Interpretation of Preclinical Evidence

Preclinical studies evaluated important mechanistic characteristics of FBC including extracellular matrix mimicry, cellular ingrowth, fibroblast proliferation, angiogenesis and collagen deposition in both in vivo and in vitro models [29, 52]. This evidence is helpful for understanding biologic mechanisms of FBC, but translation to clinical benefit for patients is limited by differences in animal and human wound healing—rodents heal primarily by wound contraction and humans by epithelization and granulation [53, 54]. Also, heterogeneity in experimental models and outcome reporting further complicates extension to clinical scenarios. Preclinical data is beneficial to understand the product, but cannot replace clinical evaluation [55].

### 4.4 Comparison With Other Biomaterials

One of the main limitations of the currently available literature is the near absence of studies comparing FBC scaffolds against other established biomaterials. Among the 28 studies included in this review, no RCT directly compared FBC with existing scaffolds in any clinical indication. The single RCT identified compared FBC to one alternative in one indication [29, 56]. One retrospective cohort by Karr and colleagues compared FBC (PriMatrix) with a living skin equivalent (Apligraf) in diabetic foot ulcers and venous stasis ulcers, reporting shorter mean time to healing with PriMatrix; however, this was a non‐randomized comparison with unequal baseline wound characteristics and no adjustment for confounders [52, 57]. In light of these studies, the literature could benefit from a RCT aimed at comparing the performance of FBC against the other alternatives already in clinical use.

The broader literature regarding biomaterials indicates that scaffold performance varies depending on source tissue, processing protocols and collagen architecture, making extrapolation from noncomparative data problematic [58]. Studies comparing bovine versus porcine derived ADMs in abdominal wall reconstruction have shown unique complication profiles despite similar recurrence rates, highlighting the importance of direct comparative studies [55]. Also, randomized trials comparing biologic and synthetic mesh in hernia repair have shown substantial differences in durability, complications and cost‐effectiveness [59]. Though these findings are based on permanent mesh rather than absorbable collagen scaffolds, they underscore the point that material selection cannot be guided by isolated efficacy data alone.

Therefore, it is extremely difficult to determine from the available evidence whether FBC scaffolds are superior compared to alternative dermal matrices or standard wound care protocols. The abundance of studies lacking concurrent control cohorts means that the wound healing rates cannot be attributed to with confidence to FBC versus natural wound healing, patient selection, or use of adjunctive therapies. Even though studies report high wound closure rates (75%–100%) and healing times as low as 4 weeks in some cohorts, the absence of comparator arms hinders conclusions regarding relative efficacy. Without direct comparative data, clinicians cannot make evidence‐based decisions regarding when FBC is beneficial over other available options, which patients will likely benefit, or if the clinical benefit, if any, justifies the additional cost.

The gap is of high importance given the difference in cost among advanced wound care products. Biologics and tissue derived scaffolds are typically more expensive than standard wound dressings, and prices vary further among biologic matrices based on product and source material [60]. In the absence of sufficient comparative data demonstrating superior outcomes, cost considerations become critical to decision making. Establishing clinical superiority or inferiority of FBC relative to alternatives is therefore necessary not only for completeness of the literature but also to ensure responsible stewardship of resources.

Above these comparative gaps, funding disclosures show a pattern that affects the entire body of evidence. Studies that show favourable outcomes for FBC disclose funding from its manufacturers, while studies that favour the comparator were funded by its manufacturers. This does not prove bias, but the presented results carry the sponsor's interest in the outcome, with no independently funded work to differentiate between the two. We say this not to negate sponsored findings, but to note the potential for bias driving the results.

### 4.5 Clinical Implications

Available evidence supports the use of FBC scaffolds as stand‐alone wound dressings and as adjunctive therapies in combination with negative pressure wound therapy, compression and autografts across a range of wound healing and reconstructive applications. Clinical studies describe successful use of FBC in cases of DFU, venous leg ulcers, pressure injuries, acute traumatic defects, burns and surgical wounds. Healing rates ranged from 75.9% to 100% across various wound types, with time to healing from 4 weeks to 6 months depending on wound characteristics and treatment protocols [57, 59, 61, 62]. FBC scaffolds demonstrated regenerative capability in preclinical studies by facilitating cellular infiltration, angiogenesis and tissue remodelling. These findings suggest that FBC represents a reasonable option within the repertoire of advanced wound care and reconstructive surgery.

### 4.6 Clinical Decision Making and Cost Considerations

The state of the available evidence is insufficient to support standardized clinical recommendations or to rightly justify broad adoption without further investigation. The lack of comparative data limits the ability to determine optimal patient selection criteria, specific clinical indications in which FBC would be preferred over alternatives, or circumstances where FBC should be used as monotherapy versus as an adjunctive therapy. Moreover, inconsistent outcome reporting and heterogeneous study populations limit the ability to predict expected healing trajectories or complication rates in specific clinical scenarios. Practitioners considering FBC scaffolds must therefore rely on clinical judgement, patient‐specific factors and institutional experience rather than evidence‐based protocols. Cost considerations further complicate decision‐making, as the economic value proposition of FBC relative to other advanced wound care products remains unclear without comparative effectiveness data [60].

This review highlights several priorities for upcoming research. First, adequately powered RCTs comparing FBC with well‐established alternatives such as ADMs, skin equivalents and standard wound care are necessary to properly evaluate comparative efficacy and safety profiles [63]. Such studies should divide analyses by wound aetiology and evaluate clinically meaningful outcomes with adequate follow‐up to assess durability of healing and recurrence rates. Second, standardization of outcome measures is essential to support future meta‐analyses and cross‐study comparisons [64]. Standardized outcomes for wound healing and reconstructive surgery should be developed and adopted in future studies to substantiate outcomes that are clinically relevant and methodologically comparable. Third, long‐term safety data including complications, degradation kinetics and immunologic responses require systematic evaluation, especially in contaminated wound environments as results differ from a sterile surgical environment.

Finally, translational research integrating preclinical mechanistic insights with clinical outcomes is needed to clarify the biologic basis for observed differences in scaffold performance and to identify biomarkers or patient characteristics that predict response to FBC therapy. Understanding the interplay between collagen composition, crosslinking density, processing methods and host tissue integration may inform next‐generation scaffold design and personalized selection strategies [52]. Until such evidence becomes available, FBC scaffolds should be considered as one option among several in wound healing and reconstructive applications, with selection guided by wound characteristics, patient factors, clinician experience and institutional resources rather than by definitive evidence of superiority.

### 4.7 Limitations

This study is not without limitations. Of the 28 identified studies, most were limited by small sample sizes. This limitation is compounded by the predominance of observational study designs. Accordingly, heterogeneity in study design and setting contributes to inconsistent outcome reporting, posing challenges to the meaningful synthesis and interpretation of results.

### 4.8 Future Directions

Future studies should include prospective trials comparing FBC to standards of care or other dermal scaffolds to address the aforementioned limitations. Existing literature could also be strengthened by the adoption of standardized outcome measures across clinical studies. Also, present studies lack long‐term safety data, which is necessary for informing clinical risk. Lastly, future investigations should consider exploring the use of FBC as a monotherapy versus an adjunct in wound and reconstruction therapy.

## 5 Conclusion

This systematic review demonstrates that FBC biomaterials have potential for broad applications in wound, regenerative and reconstructive applications. Preclinical studies demonstrate biologic plausibility and clinical studies highlight FBC's possible role in supporting chronic wound healing. Though outcome reporting was widely inconsistent, most studies denote favourable outcomes regarding the use of FBC including low inflammatory response and effective wound healing across studied indications. Despite promising data, current literature remains fragmented, thus further investigations focusing on comparing FBC with existing alternatives is necessary before a consensus can be established on FBC recommendations.

## Funding

The authors have nothing to report.

## Conflicts of Interest

The authors declare no conflicts of interest.

## Data Availability

Data sharing not applicable to this article as no datasets were generated or analysed during the current study.
